# Pregnancy outcome in long- versus short-acting gonadotropin-releasing hormone agonist cycles in participants with normal ovarian reserve: An RCT

**DOI:** 10.18502/ijrm.v21i9.14402

**Published:** 2023-10-30

**Authors:** Roya Kabodmehri, Nasrin Ghanami Gashti, Azadeh Raoufi, Marzieh Mehrafza, Zahra Nikpouri, Elmira Hosseinzadeh, Ahmad Hosseini

**Affiliations:** ^1^Reproductive Health Research Center, Department of Obstetrics and Gynecology, Al-zahra Hospital, School of Medicine, Guilan University of Medical Sciences, Rasht, Iran.; ^2^Mehr Fertility Research Center, Guilan University of Medical Sciences, Rasht, Iran.

**Keywords:** Gonadotropin-releasing hormone, In vitro fertilization, Pregnancy outcome, Ovarian hyperstimulation syndrome.

## Abstract

**Background:**

There is no agreement on which of the 2 gonadotropin-releasing hormone (GnRH) agonist protocols are the most efficient, neither there is any consensus on which one yields a better clinical pregnancy percentage.

**Objective:**

The present study aims to compare the effectiveness of reduced dosages of long- and short-acting GnRH agonists on pregnancy outcomes.

**Materials and Methods:**

In this randomized controlled clinical trial, 400 women were randomly assigned to 2 groups (n = 200/group): the reduced dosage of long-acting GnRH agonist group (group 1, 1.25 mg Decapeptyl) and the short-acting GnRH agonist group (group 2, 0.5 mg/day Buserelin Acetate). The study was conducted at Mehr Medical Institute, Rasht, Iran between July 2019 and July 2020. Biochemical and clinical pregnancy were compared between groups.

**Results:**

No significant differences were observed in the endometrial lining, the total number of retrieved and metaphase-II oocytes, progesterone, and serum estradiol levels on human chorionic gonadotropin day, fertilization rate, and top-quality embryos between the groups. The duration of induction (10.8 ± 1.7 vs. 10 ± 2.1, p < 0.001) and the total dosage of gonadotropins (2939.4 ± 945.9 vs. 2441 ± 1247.1, p < 0.001) were significantly greater in group 2 than in group 1. No significant differences were observed between the 2 groups in terms of implantation rate, chemical pregnancy rate, and clinical pregnancy rate. A higher percentage of ovarian hyperstimulation syndrome was observed in group 2 (p = 0.005).

**Conclusion:**

Due to a lower percentage of ovarian hyperstimulation syndrome in group 1 and similar assisted reproductive technology outcomes in both groups, the long protocol was found to be superior to the short protocol.

## 1. Introduction

In in vitro fertilization (IVF) cycles, controlled ovarian hyperstimulation (COH) is a crucial topic. In IVF/intracytoplasmic sperm injection (ICSI) treatment cycles, gonadotropin-releasing hormone (GnRH) agonists are employed for hypothalamic axis downregulation and ovarian suppression, followed using gonadotropins to induce controlled ovulation (1-3). Research has indicated that GnRH agonists can positively affect pregnancy outcomes in embryo transfer (ET) procedures (4).

2 types of GnRH agonist protocols, long-acting and short-acting, have been adopted in routine clinical practices. However, some studies have proposed that reducing the dose of depot GnRH agonist is associated with a decrease in the number of gonadotropins required for ovarian stimulation (5, 6).

There is no consensus on the optimal long protocol that enhances pregnancy rates in the context of the IVF procedure (7-10). This randomized clinical study aims to evaluate the effectiveness of reduced dosages of long-acting GnRH agonists compared to short-acting GnRH agonists on pregnancy rates among participants undergoing ICSI/ET treatment.

## 2. Materials and Methods

### Subjects and study design

In this randomized controlled clinical trial study, 400 participants who were candidates for ICSI at Mehr Medical Institute, Rasht, Iran, were enrolled from July 2019-July 2020.

### Study design, participants, and intervention

Women who were candidates for ICSI due to male, tubal, or unexplained factor infertility were enrolled. The inclusion criteria were as follows: age ≤ 40 yr, day 3 serum follicle-stimulating hormone (FSH) < 8 mIU/L, < 1 failed ICSI cycle, non-PCOS participants, absence of azoospermia, and no moderate to severe endometriosis. Exclusion criteria consisted of no response to ovulation induction, ET, and a bloody ET. The participants were randomly divided into 2 groups: the decreased dosage of long-acting GnRH agonist group (group 1, n = 200, Triptorelin Acetate (DecapeptylⓇ 1.25 mg, Ferring Co., Germany)) and the short-acting GnRH agonist group (group 2, n = 200, Buserelin Acetate (Cinnafact, CinnaGen, Iran)). Of the initial groups, 26 participants from group 1 (n = 174) and 48 participants from the group 2 (n = 152) were excluded from the analysis. Baseline levels of anti-Müllerian hormone (AMH) (ELISA Kit, PishtazTeb Diagnostics, Iran), FSH (ELISA Kit, PishtazTeb Diagnostics, Iran), and luteinizing hormone (LH) (ELISA Kit, PishtazTeb Diagnostics, Iran) were measured in all participants. Both groups received oral contraceptive pills (ethinyloestradiol 30 μg + levonorgestrel 150 μg) from the 3 ${}^{\text{rd}}$ to the 21 ${}^{\text{st}}$ day of menstruation.

In group 1, depot GnRH agonist (1.25 mg) was administered in the mid-luteal phase (day 21) of the preceding menstrual cycle. In group 2, participants received subcutaneous injections of 0.5 mg/day of Cinnafact starting from day 21. They continued at a reduced dosage of 0.25 mg from day 2 of the subsequent cycle until the day of human chorionic gonadotropin (hCG) injection. For both groups, ovarian stimulation was conducted using recombinant FSH (Cinnal-f, CinnaGen, Iran) and menotropins (PD HOMOG, Pooyesh Darou, Iran). Ovarian response was monitored using transvaginal ultrasonography and measurements of serum estradiol and progesterone levels. Once at least 2-3 follicles with sizes ranging from 18-20 mm were observed by transvaginal ultrasonography, 10,000 IU hCG (PD PREG, Pooyesh Darou, Iran) was administered intramuscularly. Oocyte retrieval via sonography was performed 34-36 hr after triggering final oocyte maturation.

### ICSI, embryo culture, and transfer

ICSI procedures were carried out 4-6 hr following oocyte retrieval. Fertilization was identified once 2 polar bodies and 2 pronuclei were detected 16-18 hr after sperm injection. The quality of embryos was assessed and graded using Gardner's scoring system (11). Under normal conditions, ET was conducted 3-5 days after fertilization. However, in cases of improper endometrial thickness and ovarian hyperstimulation syndrome (OHSS), embryos were cryopreserved.

During ET, participants received a daily dose of 400 mg vaginal suppository of progesterone (Cyclogest 400 mg, Alpharma, England) starting on the day of oocyte retrieval and continuing until the 10 ${}^{\text{th}}$ wk after the transfer to support the luteal phase.

### Outcome measurement 

The primary endpoints for this study were defined as the rate of biochemical pregnancy (serum beta human chorionic gonadotropin level exceeding 25 mIU/mL, β-hCG ELISA kit, DiaZist, Iran) and clinical pregnancy (detection of a gestational sac with a heartbeat on ultrasound). Secondary endpoints included the dosage of gonadotropins and the duration of ovarian stimulation. Additional parameters evaluated in the study included progesterone levels on hCG day (ng/ml), estradiol levels on hCG day (pg/ml), endometrial thickness (mm), total number of retrieved eggs, total number of mature metaphase-II (MII) eggs, fertilization rate (2PN embryos/MII), number of top-quality embryos, implantation rate, incidence of OHSS, and cancellation percentage (%).

### Sample size

The sample size of participants was determined based on the difference in clinical pregnancy, which was the primary endpoint, as reported in the study by Mao et al. (12). The study was designed with 90% statistical power, a type I error rate of 0.05, and an anticipated difference of 16.2% between the 2 groups (59.6% clinical pregnancy rate in the long-acting GnRH group compared to 43.4% in the short-acting GnRH group).

### Blinding

Each participant was assigned a unique code, which the researcher placed inside sealed envelopes. Similarly, medications were enclosed in sealed envelopes, each labeled with a distinct code. Both gynecologists and participants remained blinded to the group assignments.

### Randomization

Block randomization techniques were employed using online software (closed pocket) to generate random numbers for the randomization process. An allocation ratio of 4:4 was utilized. Opaque and sealed envelopes, each containing the allocation information, were provided to the gynecologists.

### Ethical considerations 

This research was permitted by the Ethics Committee of the Guilan University of Medical Sciences, Rasht, Iran (Code: IR.GUMS.REC.1398.094) and is registered on the IRCT website, which has been updated on 20 June 2023. A consent form was obtained from all participants before the ovarian stimulation treatment.

### Statistical analysis

Data analysis was done using version 23.0 of SPSS Inc., Chicago, Illinois, USA (SPSS). The results were presented as mean ± SD (standard deviation), median (min, max), or percentage, as appropriate. The normality of distribution was assessed using the Kolmogorov-Smirnov test. For parametric variables, Student's *t *test was utilized, while the Mann-Whitney test was applied for non-parametric variables. Categorical variables were analyzed using the Chi-square test. A p-value of < 0.05 was considered statistically significant.

## 3. Results 

**Figure 1:**
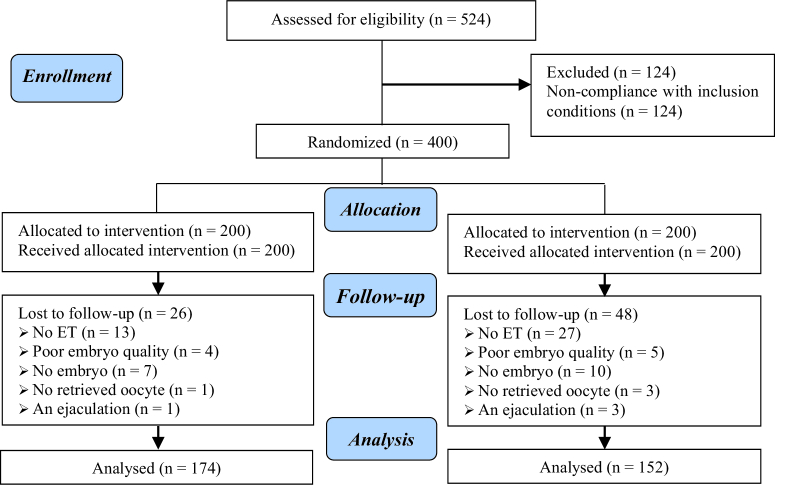
CONSORT flow diagram.

A total of 400 participants were initially assigned to 2 groups, with 200 individuals in each group. However, 74 participants were subsequently excluded from the analysis: 26 from the long-acting GnRH agonist group (no ET (n = 13), poor embryo quality (n = 4), no embryos (n = 7), no retrieved oocytes (n = 1), anejaculation (n = 1)), and 48 from the short-acting GnRH agonist group (no ET (n = 27), poor embryo quality (n = 5), no embryos (n = 10), no retrieved oocytes (n = 3), anejaculation (n = 3)) (Figure 1).

Baseline characteristics of the participants are presented in table I. No significant differences were observed in age, body mass index, basal AMH, FSH, LH levels, and duration of infertility. The results of assisted reproductive technology (ART) procedures are summarized in table II. No significant differences were observed between the groups in terms of endometrial thickness, total number of retrieved and MII oocytes, progesterone and estradiol levels on hCG day, fertilization rate, and top-quality embryos. However, a significant difference was observed in the duration of ovarian stimulation and the overall gonadotropin dosage (p < 0.001). Notably, there was a significantly higher cancellation rate in group 2 (p = 0.007).

No significant differences were observed between the 2 groups in terms of implantation rate, chemical pregnancy rate, and clinical pregnancy rate. However, group 2 exhibited a significantly higher rate of OHSS (p = 0.005) (Table II, III).

**Table 1:** Evaluation of basal characteristics of participants between group 1 and group 2


**Variables **	**Group 1 (n = 174)**	**Group 2 (n = 152)**	**P-value**
Age (yr)*	30.3 ± 4.8	30.2 ± 5.14	0.83
BMI (Kg/m^2^)*	25.9 ± 4	25.7 ± 3.6	0.65
AMH (ng/ml)**	2.5 (0.29-9)	2.7 (0.06-10)	0.21
FSH (mIU/ml)*	5.1 ± 1.7	5.2 ± 1.7	0.75
LH (mIU/ml)**	3.3 (0.9-10.4)	3.4 (1.1-12)	0.61
Duration of infertility*	6.1 ± 5.2	5.3 ± 4.8	0.29
*Data presented as Mean ± SD. Student's *t* test. **Data presented as median (min-max). Mann-Whitney test. BMI: Body mass index, AMH: Anti-Mullerian hormone, FSH: Follicle-stimulating hormone level on day 3 of cycle, LH: Luteinizing hormone on cycle day 3			

**Table 2:** Evaluation of ART outcome between group 1 and group 2


**Variables **	**Group 1 (n = 174)**	**Group 2 (n = 152)**	**P-value**
Stimulation duration (day)*	10.8 ± 1.7	10 ± 2.1	< 0.001
Total dose of gonadotropins (IU)*	2939.4 ± 945.9	2441 ± 1247.1	< 0.001
Progesterone level in hCG day (ng/ml)*	1.11 ± 0.2	0.98 ± 0.2	0.31
Estradiol level in hCG day (pg/ml)*	2511.9 ± 2	3162.3 ± 2	0.08
Endometrial line (mm)*	8.7 ± 1.3	8.6 ± 1.2	0.50
Number of picked-up oocytes*	12.9 ± 7.4	13.2 ± 7.7	0.72
Number of MII oocytes*	10.6 ± 6.7	10.97 ± 6.5	0.58
Fertilization (2PN embryos/MII)*	0.74 ± 0.3	0.73 ± 0.3	0.80
Number of highest-quality embryos*	4.1 ± 2.9	4.4 ± 3.05	0.38
Implantation rate**	59 (33.9)	55 (36.2)	0.72
OHSS rate**	12 (6.9)	26 (17.1)	0.005
Cancellation rate**	26 (13)	48 (24)	0.007
*Data presented as Mean ± SD. Student's *t* test. **Data presented as n (%). Chi-square test. ART: Assisted reproductive technology, 2PN: 2 pronuclear, MII: Metaphase II, OHSS: Ovarian hyperstimulation syndrome, hCG: Human chorionic gonadotropin			

**Table 3:** Evaluation of chemical and clinical pregnancy rate among groups with per-protocol and intention-to-treat analysis


**Variables**		**Group 1 (n = 174)**	**Group 2 (n = 152)**	**P-value**
**Chemical pregnancy**				
	**Per-protocol**	63/174 (36.2)	64/152 (42.1)	0.36
	**Intention-to-treat**	63/200 (31.5)	64/200 (32)	0.19
**Clinical pregnancy**				
	**Per-protocol**	59/174 (34)	56/152 (37)	0.24
	**Intention-to-treat**	59/200 (29.5)	56/200 (28)	0.56
Values were shown as number (%). Chi-square test				

## 4. Discussion 

The results of the present study suggest that both groups exhibited similar outcomes in assisted ART, but with a lower incidence of OHSS in group 1. This indicates that the long-acting protocol may be superior to the short-acting protocol.

The optimal protocol for ovarian stimulation in IVF cycles has been a topic of debate. Various COH protocols involving different types, doses, and timing of gonadotropins or GnRH analogs have been studied to reduce cycle cancellation rates and ovarian suppression (13-15). The long protocol, which initiates downregulation in the early follicular or mid-luteal phase preceding IVF treatment, has shown promising results such as decreased cycle cancellation rates, prevention of early luteinization and endogenous LH release, increased follicle development, and improved clinical pregnancy rates (16, 17). In contrast, the short-acting GnRH agonist requires daily low-dose injections starting in the middle of the luteal phase of the previous cycle and continuing until the day of hCG administration, making it less convenient for participants. The long protocol is more user-friendly, requiring only a single depot dosage of GnRH agonist in the middle of the luteal phase of the preceding cycle. However, its disadvantages include the need for more gonadotropins, longer usage periods, and consequently higher costs for IVF treatment (10).

In our study, we analyzed 2 COH protocols: the long-acting form of 1.25 mg Triptorelin and the short-acting form of 0.5 mg Buserelin, both in normal responders. The clinical pregnancy rate did not show a significant difference between the 2 groups.

In a study by Wu et al., the influence of different GnRH agonist administration methods on pregnancy outcomes in IVF-ET participants were evaluated. They retrospectively analyzed data from 5217 participants and categorized them into groups based on their pituitary suppression. Our findings did not reveal significant differences between the 2 groups in terms of egg numbers, MII oocyte counts, fertilization rates, cleavage rates, and blastocyst formation rates. However, the clinical pregnancy rate and embryonic implantation rate were significantly higher in group 1 compared to group 2. The use of depot GnRH agonists was found to effectively reduce cancellation rates and improve clinical pregnancy outcomes in fresh treatment protocols (18).

Contrary to our study, a research concluded that long-acting GnRH agonists could increase the clinical pregnancy rate in IVF-ET patients. Additionally, another study reported a significantly higher live birth rate in the long-acting GnRH agonist group compared to the short-acting group (9).

In a study, the total number of retrieved eggs, the number of MII eggs, fertilization rates, and embryo quality were found to be similar between the 2 groups (19). This finding contrasts with a study in Nigeria, which indicated significantly higher numbers of retrieved oocytes and fertilization rates in the long-acting GnRH agonist group (7).

Duan et al. compared the effectiveness of 2 forms of GnRH agonists in clinical outcomes of IVF/ICSI long protocol cycles. They concluded that the depot agonist was associated with higher levels of gonadotropins and a longer duration of usage for induction of ovulation. However, this could lead to increased expenses and potentially negative impacts on pregnancy outcomes due to a higher incidence of OHSS and reduced rates of good-quality embryo development and implantation (20).

Consistent with previous reports, our study found that the long-acting GnRH agonist group had a longer stimulation period and a higher gonadotropin dosage compared to the short-acting group (18, 20).

Our research revealed a significantly higher OHSS rate in the short-acting GnRH agonist group compared to the long-acting group, which contrasts with the previous report indicating a significantly higher OHSS rate in participants who underwent long-acting GnRH agonist treatment (20).

## 5. Conclusion

As discussed in the previous sections, the lower incidence of OHSS observed in group 1, coupled with comparable ART outcomes between the 2 groups, suggests that the long-acting depot protocol holds an advantage over the daily agonist protocol. This superiority is attributed to the greater comfort it offers to participants. The management of OHSS presents significant challenges, underscoring the importance of identifying improved ovarian stimulation methods to either prevent OHSS or mitigate its occurrence. Our study's findings, which demonstrated a reduction in the OHSS rate within the long-acting group, contribute valuable insights to the ongoing research in this area. By highlighting the potential of the long-acting protocol in addressing OHSS, this study may pave the way for further advancements in ovarian stimulation techniques that prioritize participants comfort and safety. Such insights are crucial in refining IVF protocols and enhancing participant experiences in ART.

##  Conflict of Interest

The authors declare that there is no conflict of interest.
